# Implementation outcomes of Humanwide: integrated precision health in team-based family practice primary care

**DOI:** 10.1186/s12875-021-01373-4

**Published:** 2021-02-02

**Authors:** Cati G. Brown-Johnson, Nadia Safaeinili, Juliana Baratta, Latha Palaniappan, Megan Mahoney, Lisa G. Rosas, Marcy Winget

**Affiliations:** Division of Primary Care and Population Health, Stanford University School of Medicine, Stanford MSOB, 1265 Welch Rd x216, Palo Alto, CA 94305 USA

**Keywords:** Precision health, Pharmacogenomics, Genetic testing, Digital health, Implementation science, Primary care, Mixed methods

## Abstract

**Background:**

Humanwide was precision health embedded in primary care aiming to leverage high-tech and high-touch medicine to promote wellness, predict and prevent illness, and tailor treatment to individual medical and psychosocial needs.

**Methods:**

We conducted a study assessing implementation outcomes to inform spread and scale, using mixed methods of semi-structured interviews with diverse stakeholders and chart reviews.

Humanwide included: 1) health coaching; 2) four digital health tools for blood-pressure, weight, glucose, and activity; 3) pharmacogenomic testing; and 4) genetic screening/testing. We examined implementation science constructs: reach/penetration, acceptability, feasibility, and sustainability. Chart reviews captured preliminary clinical outcomes.

**Results:**

Fifty of 69 patients (72%) invited by primary care providers participated in the Humanwide pilot. We performed chart reviews for the 50 participating patients. Participants were diverse overall (50% non-white, 66% female). Over half of the participants were obese and 58% had one or more major cardiovascular risk factor: dyslipidemia, hypertension, diabetes. Reach/penetration of Humanwide components varied: pharmacogenomics testing 94%, health coaching 80%, genetic testing 72%, and digital health 64%. Interview participants (*n*=27) included patients (*n*=16), providers (*n*=9), and the 2 staff who were allocated dedicated time for Humanwide patient intake and orientation. Patients and providers reported Humanwide was acceptable; it engaged patients holistically, supported faster medication titration, and strengthened patient-provider relationships. All patients benefited clinically from at least one Humanwide component. Feasibility challenges included: low provider self-efficacy for interpreting genetics and pharmacogenomics; difficulties with data integration; patient technology challenges; and additional staffing needs. Patient financial burden concerns surfaced with respect to sustainability.

**Conclusion:**

This is the first report of implementation of a multi-component precision health model embedded in team-based primary care. We found acceptance from both patients and providers; however, feasibility barriers must be overcome to enable broad spread and sustainability. We found that barriers to implementation of precision health in a team-based primary care clinic are mundane and straightforward, though not necessarily easy to overcome. Future implementation endeavors should invest in basics: education, workflow, and reflection/evaluation. Strengthening fundamentals will enable healthcare systems to more nimbly accept the responsibility of meeting patients at the crossroads of innovative science and routinized clinical systems.

**Supplementary Information:**

The online version contains supplementary material available at 10.1186/s12875-021-01373-4.

## Background

Precision medicine holds promise for revolutionizing care delivery, and precision health takes precision medicine even closer to the goal of patient-centered health care. Precision health connotes a shift of precision medicine from individualized treatment and risk stratification to disease prevention and wellness promotion alignment with patient-focused goals [1]. These overarching goals are well-suited for the generalist approach to primary care. Reports of implementation outcomes related to integration of precision health into health care delivery, however, have been limited to condition-specific care protocols [2]. While there is recognition that implementation of precision health in primary care is needed, to our knowledge no comprehensive precision health package embedded in primary care has been reported.

There is a dearth of information to inform implementation of novel, complex, or multilayered interventions. Implementation science research, with its focus on understanding *how* and *why* change is successfully or unsuccessfully enacted, is crucial for healthcare systems to integrate cutting-edge approaches into live healthcare delivery. Pragmatic evaluation of the implementation processes of early pilots has been recommended specifically for precision medicine to highlight insights for troubleshooting future adaptation and spread [3].

In January 2018 Stanford Health Care launched Humanwide, a pilot embedding multifaceted precision health into a team-based primary care setting, to assess feasibility and identify facilitators and barriers to spreading the model. Humanwide is a precision health initiative aimed at leveraging high-tech and high-touch medicine to promote wellness, predict and prevent illness, and tailor treatment to individual biomedical and psychosocial needs [4]. We assessed implementation outcomes, specifically *penetration/reach*, *acceptability*, *feasibility*, and *sustainability* [5] to inform future implementation initiatives and facilitate scale/spread of precision health in primary care. We also assessed early potential clinical benefit to patients.

## Methods

### Humanwide model

Humanwide [4] builds on culminating precision health evidence related to health coaching [6], digital health [7], pharmacogenomics [8], and genetic testing [9]. Due to the multiple components, the designers predicted that team-based care would be necessary for successful implementation. The Stanford implementation clinic for Humanwide was well-versed in practicing team-based care (Primary Care 2.0), which includes typical MD and Advance Practice Provider (NP or PA) health professionals, as well as a diabetes pharmacist, dietician, mental health provider, and triage nurse [10].

The goal of Humanwide was to provide personalized care at all points of the care continuum: wellness, prevention, treatment, chronic disease management, and complex case management. Components of Humanwide included: 1) Health coaching- a one-hour wellness assessment with primary care providers (MD or APP) trained in health coaching to establish patient-stated goals and collect social determinants of health with follow-up visits in person or by phone; 2) Digital health - four wireless tools that connect patient values taken at home to the clinic electronic health record (EHR) for blood-pressure (cuff), weight (digital scale), glucose (glucometer), and activity (smartphone pedometer); 3) Pharmacogenomics using saliva-based screening for drug-gene interactions; and 4) Genetic screening to assess risk of Centers for Disease Control (CDC) Tier 1 markers of hereditary breast and ovarian cancer syndrome, Lynch syndrome, or familial hypercholesterolemia [9]. Support for these components included training for health coaching, a two-hour orientation for pharmacogenomic testing which included healthcare worker testing, and in-depth workflow conversations with genetic counselors.

Pharmacogenomics supports tailored medication decisions, including how medication may interact with an individual’s genetically-predetermined metabolic pathways and how medications may interact with one another in a multi-pharmaceutical situation. Data from pharmacogenomic assessment can be relevant in any treatment situation, but were anticipated to be particularly useful for patients with chronic diseases or complex cases who might have multi-pharma needs.

Humanwide was implemented in a single academic primary care clinic in the community over an approximate 12-month period. Patients were invited to participate by their primary care providers. There were not specific inclusion/exclusion criteria, rather there was a goal of recruiting a broad range of patients with respect to health complexity, race/ethnicity, and age. All devices, pharmacogenomics and genetic tests, and associated consultation/counseling were provided free to participants in order to alleviate potential cost issues that might interfere with full participation.

### Ethics statement

All clinical standards of care were followed in this QI initiative project. Humanwide was designed to take the best existing personalized care tools and integrate them into primary care. These approaches (1. home-monitoring of glucose, blood pressure, weight, physical activity; 2. pharmacogenomic testing; 3. genetic testing; 4. health coaching) are currently offered by licensed physicians. All existing guidelines for the ordering, interpretation, and follow up of these approaches were implemented in this pilot. For home-monitoring, the clinic created dashboards and alerts to help monitor individual values, medical assistants were trained health coaches, and the onsite dietician and diabetes pharmacist served as part of the care team. For pharmacogenomic data, our onsite pharmacist obtained additional training and certification. All medication recommendations were reviewed with a physician with expertise in pharmacogenomics prior to communication with the primary care team. All patients underwent pre-test and post-test genetic counseling with a certified genetic counselor as current guidelines recommend.

This quality improvement evaluation was deemed not to be human subjects research by the Stanford Institutional Review Board, protocol #43279.

### Reflexivity statement

The Evaluation Sciences Unit at the Stanford School of Medicine is intentionally an external evaluation partner for Stanford Health Care and other healthcare organizations. We operate as a third-party evaluator, outside healthcare delivery or administration. Our duty as evaluators working within the quality improvement format is to provide unbiased high-quality evaluation methods and interpretation. Our team works independently from funders (eg. in this instance the Dean’s Office) and in partnership with clinician implementers (including co-author Dr. Mahoney). As the objective third-party, the Evaluation Sciences Unit conducted and interpreted the interviews, which could have been more easily biased by interested parties in collection or analysis. Clinical partners conducted the chart review, which relies on more objective EHR data.

To ensure independence, our manuscript drafts are not reviewed by funders. As a leader in high-quality evaluation of quality improvement, our strategy is to provide feedback early and often so that implementers can address issues and provide best possible care for patients. We intentionally provide a critical research lens focusing on unbiased methods and data interpretation. We do partner closely with clinical implementers in our evaluations, because clinical input in these types of studies is critical to context-specific insights.

### Data collection

We obtained data related to implementation outcomes of penetration/reach, acceptability, feasibility, and sustainability via chart review and semi-structured qualitative interviews at initial enrollment and approximately 6–9 months post enrollment. Interviews were conducted with 16 participating patients, 9 providers, and the 2 staff who were allocated dedicated time for Humanwide patient intake and orientation. Interviews were audio-recorded and transcribed. Chart reviews captured demographic information (age, gender, race/ethnicity, occupation), participation in Humanwide components, and preliminary clinical outcomes relevant to Humanwide for all 50 patients.

Semi-structured interviews captured feasibility and acceptability of Humanwide, and facilitators/barriers to inform future scale/spread. Interview guides for providers and patients were informed by qualitative guides from previous studies in precision health [11, 12] as well as implementation science frameworks such as the Consolidated Framework for Implementation Research (CFIR) [13]. Topic areas included: 1) perception and experience with precision health, 2) positive or negative impacts of precision health on health and wellbeing, 3) barriers or facilitators to sustainability, and 4) perceptions of the potential to impact health disparities (see Additional file 1 and 2, interview protocols for providers and patients respectively).

### Data analysis

Consensus qualitative analysis improves the quality of coding and decision-making in diverse groups where majority views can be queried [14]. To ensure optimal consensus, we used a three-step process to integrate inductive and deductive approaches [15], in alignment with Richards and Hemphill 2017 [16]. First, we used NVivo 12 to capture excerpts relevant to each Humanwide module - health coaching, digital health, pharmacogenomics, and genetic screening. Second, using queries, we produced excerpt collections organized by module. Third, we identified and iteratively refined emergent themes and discussed with all co-authors. These emergent themes were then mapped to implementation science constructs *penetration/reach*, *acceptability*, *feasibility*, *sustainability* based on Procter, et al. [5], which were chosen to appropriately align with the early pilot phase of Humanwide.

## Results

### Participant demographics

Fifty of 69 patients (72%) invited by their primary care provider participated in the Humanwide pilot. Participants were diverse overall (Table 1) with 50% non-white and 66% female. Over half of the participants were obese and 58% had one or more of the following major cardiovascular risk factors: dyslipidemia, hypertension, diabetes. About one quarter of participants were employed by the technology sector (*n*=12, 24%).

**Table 1:** Patient characteristics of precision health pilot participants

Patient Characteristics (***n***=50)	
**Age in years**	
Median (range)	47 (24–86)
**Gender**	
Woman	33 (66%)
**Race/Ethnicity**	
White	25 (50%)
Asian	13 (27%)
Hispanic	9 (19%)
African American	3 (6%)
**Clinical conditions**	
Cardiovascular Risk	29 (58%)
BMI > 30	26 (52%)

#### Implementation outcomes

##### Penetration

Overall reach/penetration of Humanwide was low with only 21 of 50 (42%) patients receiving all four components. The reach/penetration of Humanwide components ranged from moderate to high: 94% (46 of 50) accessed pharmacogenetic testing; 80% (40 of 50) accessed health coaching with an average of 2+ sessions; 72% (36 of 50) accessed genetic testing; and 64% (32 of 50) participated in digital health (Table 2). All patients completed a wellness assessment to capture family medical history and health behavior data. This information was needed to assess appropriate next steps for genetic testing and health coaching, respectively. Initially, enrollees were offered all four components regardless of medical background and interest. However midway through the implementation (starting at patient #22 of 50), the selection of individual components was tailored to the patient’s medical risk profile and preference.

**Table 2:** Implementation outcomes for each component of the Humanwide precision health pilot

Implementation Outcomes	Pharmacogenomics	Health coaching	Genetic testing	Digital health
**Penetration/reach** Overall 42% (21 of 50)	94% (46 of 50)	80% (40 of 50) average of 2+ sessions	72% (36 of 50)	64% (32 of 50)
**Acceptability** (satisfaction, agreeable, palatable with it) *what is acceptable*	Positive: Patients and providers very positive; perceived as relevant to health Negative: Specialist resources needed to interpret results	Positive: Engaged patients holistically; interested patients reported value and potential to address health disparities Negative: Not all patients interested	Positive: Driver for patient participation in Humanwide Negative: Variants of uncertain significance^a^	Positive: Helped patients meet health goals; supported strong patient-provider relationships; quicker time to titrate meds Negative: Data and communication volume create burden for providers
**Feasibility** (suitability for everyday use, can be carried out, resources/training/ Staff/self-efficacy)	Positive: Successfully executed Barriers: Primary care physicians cannot interpret results; requires specialist; data integration not standardized in EHR	Positive: Successfully executed Negative: Availability of resources	Positive: Successfully executed Negative: Requires specialist/ additional training to interpret results; data integration not standardized; specialist access limited	Positive: Integrated into regular patient-provider communication Negative: Technology challenges – Bluetooth pairing
**Sustainability/ Spread**: Issues that facilitate or threaten spread/scale	Specialist resources needed to interpret results; insurance coverage/cost	Time (providers and patients) and skills needed (providers and staff)	Specialist resources needed to interpret results; insurance coverage/cost	Patient maintenance of digital health monitoring did not occur

^a^Variants of uncertain significance include incidental findings that are not actionable and are not interpretable based on the current state of the science [17]

##### Acceptability

Humanwide was acceptable to patients, with few caveats (Table 2). Patients perceived that pharmacogenomics was the most novel aspect of Humanwide and reported anticipation that the service would greatly impact their care in the future. Of those interviewed, 5 of 10 patients tested found the pharmacogenomic results important to their health.

#### Health coaching

Health coaching engaged patients holistically, and patients reported a highly positive experience overall. For those interviewed who accessed health coaching (*n*=8), the service was seen as an asset, providing accountability and guidance in reaching health goals. Patients suggested that health coaching could bridge the gap between provider recommendations and patient adherence: “Before [Humanwide], you would go to a regular doctor and the doctor says, ‘Hey, you need to lose weight’ … a health coach can say okay what are you doing, how are you doing, and then try to work within your routine to provide some alternatives to be beneficial to me health-wise.” Patients viewed the personalized, big-picture nature of health coaching to have potential to address health disparities resulting from social determinants of health such as access to transportation, food, and housing, “because everyone lives a little bit differently.”

#### Genetic testing

Patients with complex personal and family medical histories were highly interested in genetic testing and many reported participating in the pilot specifically to receive it. Patients who were interviewed reported feeling reassured by genetic testing results, “When my daughter got leukemia, I just wanted to know is there any genetic connection?” Variants of uncertain significance (i.e., a situation where there are insufficient data linking genetic markers to health outcomes) were frustrating to both patients and providers, and perceived as a major implementation issue.

#### Digital health

The ability to track measurements with digital health devices and view them over time was helpful to patients in meeting health goals. Patients reported that digital health also enhanced a sense of partnership and accountability with the entire healthcare team: “I feel like I have outside eyes having access to my data, especially my PCP [primary care provider], so I want to make sure I get my steps and do all that. I’m actually consciously doing stuff.” Providers endorsed that data from digital health in Humanwide “changes the conversation,” and can speed medication adjustments, strengthen relationships, and even increase adherence: “It’s huge [digital health data for blood pressure management] … we’re able to titrate them quicker … we get them to goal quicker. We have a tighter relationships …”.

Data review and patient follow-up through digital health, however, was reported to be an additional burden for providers and staff. Providers suggested that the burden could be lessened by setting expectations that intensive remote monitoring via digital health is only for time-limited interventions such as medication titration: “That’s what’s so overwhelming, people expect that I’m going to do it forever. I just really want to know for the next couple of months. I want to get you under control, and then you’re off … am I not teeing it [expectations] up correctly?”

##### Feasibility

Humanwide uncovered important challenges for feasibility of implementation in a team-based care environment (Table 2). Primary barriers included: 1) low provider self-efficacy in interpreting genetic/pharmacogenomic tests; 2) poor integration of patient data into EHR and workflows for all Humanwide components; 3) patient difficulty with usability of technology, for example Bluetooth pairing in digital health; and 4) lack of staffing for digital health engagement and health coaching. Despite these barriers, activities supporting each of the modules of Humanwide were completed to some extent, indicating feasibility at a foundational level.

#### Pharmacogenomics

Providers and staff reported low self-efficacy in interpreting pharmacogenomic results due to limited training and emphasized that a specialist is needed to interpret and communicate findings to patients. Another challenge was that existing pharmacogenomic databases are limited in scope which resulted in inconclusive test results for several patients. This limitation was identified early on and prompted providers to recommend limiting pharmacogenomic testing to patients who might clearly benefit, such as those with multiple medications or those preparing for an operation. Integration of pharmacogenomic results into the EHR was also a challenge, due to the lack of a clearly defined section in the EHR to import or document test results. Some more significant pharmacogenomic test results were documented in the medication allergies section of the EHR while others documented it elsewhere or not at all.

#### Genetic testing

Similar to pharmacogenomics results, providers reported a gap in workflows and EHR structures for storing and accessing genetic test results, as well as a need for additional training in interpreting results. Genetic testing feasibility was also hindered by limited access to genetic counselors, which led to longer wait times for patients to receive results.

#### Digital health

Technological glitches impaired use of digital health tools among patients. Bluetooth pairing between digital scales, blood pressure cuffs, glucometers, and pedometers and patient devices was a serious barrier to implementation and required significant time from staff to troubleshoot. One hour of a medical assistant’s time was needed to set up the technology for each patient and sync digital health tools. Even with this support, some highly tech-savvy patients struggled to overcome technical issues, most often due to difficulties with Bluetooth pairing.

##### Sustainability

Sustainability concerns echoed many of the feasibility issues for Humanwide (Table 2). Additionally, cost was noted as a potential future barrier (technology and extra clinical visits were free to Humanwide participants). Some patients reported that if they had been required to pay for services out-of-pocket, they would not have participated in genetic/pharmacogenomic testing. There was a stated preference from providers and patients for the health coaching and engagement with digital health to be combined. However cost in terms of time was also identified as a barrier for the sustainability and spread of health coaching within the primary care team for both providers and patients. To address the time concern, providers suggested using care team members or telehealth/online health coaching in the future.

#### Clinical outcomes

##### Overall

Based on our chart review, care plans were adjusted for all patients, signaling that patients may have clinically benefited from at least one component of Humanwide. Participants’ care plan adjustments included one or more of: tailored medication from pharmacogenomics; appropriate genetic testing; and/or early identification of risk through remote digital health monitoring.

##### Pharmacogenomics

Eleven patients (22%) had an immediately-actionable medication finding, related to post-operative opioids, statins, anti-depressants, or anti-ulcer medications, resulting in 18 drug management changes: 7 dose changes, 4 medication changes, and 7 alternative initial medications selected. Additionally, among the 16 patients who reported current or past adverse effects to medications, 9 of 23 of their adverse effects were attributable to genetic impact on their ability to metabolize certain medications, i.e., their pharmacogenomic metabolizer status.

##### Health coaching

Patient care plan goals focused on lifestyle behaviors such as weight, exercise, and diet. Specific patient-stated health goals commonly pertained to weight loss, exercise, diet, blood pressure regulation, blood sugar/A1c management, sleep goals, stress management, mental health, and substance use management. Prediabetes and/or prehypertension were detected in four patients during the pilot; all were able to reach their goal of controlling their glucose and blood pressure through self-management supported by health coaching.

##### Genetic screening and testing

The genetic screening tool developed for the pilot identified 10 patients (29%) with an indication for genetic testing that had not been previously identified. Although none of these patients received positive results for any of the three Centers for Disease Control Tier 1 conditions (i.e., BRCA, familial hypercholesterolemia, or Lynch disease), five of them were recommended to receive enhanced breast cancer screening based on their family history.

##### Digital health

Twenty-three of the 32 patients (72%) that used at least one digital health tool had medication adjustments as a result of participating in the digital health component. Seven specialists outside of the pilot also incorporated digital health results in their patient management (2 endocrine and 5 cardiology).

## Discussion

### Acceptability/value of Humanwide

Despite calls for exploration of integrated precision health in primary care [18], outside of individual components such as digital health or genetic/genomic testing [19], a multi-component precision health intervention has not been previously integrated into primary care practices, in part due to implementation challenges. Overall, patients reported good acceptability of Humanwide, with positive patient health outcomes. Providers and staff expressed perceptions that Humanwide supported more immediate and tailored care. Challenges for all stakeholders revolved around cost, time, provider education and training, uncertainty of genetic testing results, and clinic/staff resources.

These challenges, specifically resources/data integration, training/self-efficacy, and access to genetics and pharmacogenomics expertise, are typical challenge areas for healthcare system implementations [20, 21], and perhaps should have been addressed prior to implementing Humanwide. Several of these issues can be overcome for precision health by optimizing resources and workflows of clinical teams, for instance by training medical assistants in health coaching to support digital health integration [20]. Close partnerships with genetics experts to fully integrate genomics into primary care are also needed; a shortage of genetic counselors has been previously documented [21]. To address this critical limitation to potential spread, access to genetics counseling services could be improved through the provision of online educational materials. Another approach previously suggested in the literature would be reliance on alternative modes for visits, such as virtual video or phone visits, rather than in-person [10]. EHR integration with clinical decision support also will be necessary for sustainable implementation of a pharmacogenomics program in primary care.

### Spread and scalability

Adoption was successful among patients, primary care providers, and specialists. However, for spread and scalability, how to leverage team-based care, improve technology integration, and understand cost-effectiveness would be future research interests. Teaming and coordination needs are located both *within* primary care (eg. for digital health engagement and related health coaching) and *external to* primary care, specifically, with specialists to support pharmacogenomic and genetic testing. In addition to integrating additional external team members, technology integration needs to be improved for both patient device integration and for data and workflow integration to support clinical care. Costs are rapidly reducing for genetic and pharmacogenetic testing; however it is still not clear who (health plans, employers, or individual patients) will bear the costs of this service in the future. Even in a relatively high-resource setting (census median income in Santa Clara in 2017: $119,035 [22]), some patients balked at the idea of paying out of pocket for Humanwide and identified cost as a primary barrier to diversity and broad access to precision health care. Finally, improved technology, and possibly recommendations from behavior change literature such as leveraging gamification in digital health apps [23], are needed to facilitate facile adoption of technology as well as ongoing engagement required to actualize precision health.

### Limitations

The pilot was conducted at a single academic primary care clinic in the community, which may limit generalizability. Costs were covered by the pilot, so we were not able to directly assess potential issues related to cost and/or insurance coverage. We also note that thematic saturation may not have been reached in all topics as sample sizes were relatively small. As a first pilot project, however, initial insights on implementation outcomes provide a good starting point for next steps towards testing integration of precision health into primary care settings in the future. Finally, patients’ new ability mid-course to pick and choose components may have shifted participation choices. While we report on reach/penetration across these approaches, we acknowledge that we have not examined the difference between these two approaches in terms of reach. Our future work will engage patients in the manner this pilot shifted to by individual component instead of providing Humanwide to patients as a required total package.

## Conclusions

In summary, Humanwide shows early evidence of acceptability and clinical benefit, indicating that it is worth addressing barriers – including insurance coverage and cost – to bring precision health to patients on a larger scale. On the cusp of a new type of medicine that is personalized and focused on prevention and wellbeing, it is critical to focus on implementation science – the *how* of integrating cutting-edge approaches into the clinical mainstream. Patient participants of Humanwide were enthusiastic about it and demonstrated evidence of enhanced care as a result of participation. Our pragmatic evaluation demonstrates that barriers to implementation of precision health in a team-based primary care clinic are mundane and straightforward, though not necessarily easy to overcome. To address provider acceptability and overall feasibility in spread/dissemination and sustainment of precision health: 1) providers and staff can be educated and more thoroughly trained, 2) workflows and EHR systems can be adapted to best support patient needs and provider wellbeing, 3) technology integration can be optimized, and 4) clinics can be appropriately staffed for the demands of a precision health panel. Future implementation endeavors should invest in these basics: education, workflow, and reflection/evaluation. Strengthening fundamentals will enable healthcare systems to more nimbly accept the responsibility of meeting patients at the crossroads of innovative science and routinized clinical systems.

## Supplementary Information

**Additional file 1.** Precision Health Provider Interview Protocol.

**Additional file 2.** Precision Health Patient Interview Protocol.

## Data Availability

The datasets used and analyzed during the current study are available from the corresponding author on reasonable request.

## Abbreviations

EHR: Electronic Health Record
BRCA: BReast CAncer Gene
PCP: Primary care provider
CDC: Centers of Disease Control
