# Supplementary material for: Implementation outcomes of Humanwide: integrated precision health in team-based family practice primary care
Source: BMC Fam Pract. 2021 Feb 2;22:28. doi: 10.1186/s12875-021-01373-4 (PMC7856755; doi:10.1186/s12875-021-01373-4)
Supplement: Supplementary file 1 — Additional file 1. Precision Health Provider Interview Protocol. [file 12875_2021_1373_MOESM1_ESM.docx]

**Precision Health Provider Interview Protocol**

| **Key Components**  Implementation  Health Coaching  Pharmacogenetics  Genetic testing  Digital health | **Interview Goals**   - Provider experience - Implementation feasibility (facilitators & barriers) - Implementation lessons learned - Understand potential for implementation of Precision Health among racial/ethnic minorities |
| --- | --- |

**Protocol**

*Precision Health*

1. Let’s start by talking about what you know about Precision Health. What does Precision Health mean to you? (1) How has your understanding of Precision Health changed over the past year?
2. What impact do you think Precision Health will have on your patients’ health outcomes? How will it impact their healthcare services? (1)

*Motivation*

1. Which parts of Precision Health were most exciting? Which parts were least interesting?
2. What are your hesitations for continuing in Precision Health? What are your motivations for continuing? (Probe: barriers for sustainability – “What might make it difficult for you to continue Precision Health?)

*Satisfaction*

1. Which parts of Precision Health were better or worse than you expected?
2. Reflecting on your experience in Precision Health, what was the most important thing you learned?
3. What was your biggest surprise in participating in Precision Health? What was your biggest disappointment?

*Daily Work*

1. How did Precision Health impact your daily work?
2. What kinds of resources did you use for Precision Health? What would you need in your practice to carry out Precision Health on a regular basis?
3. How were workflow issues addressed or resolved?
4. I know we adjusted our approach with the variants of unknown significance. Are there any other tweaks that were made along that way that were made to address emerging patient concerns?
5. Which parts of digital health worked well (thinking about the different types of data received: BP, steps, weight, glucose)? Which parts of digital health did not work well?
6. Can you walk me through the visualization of the digital health data you received?
   1. What do you like about the process of receiving patient data in real-time? What don’t you like?
   2. Which pieces of the data are actionable?
   3. What kind of expectations did receiving this data place on you?

*Implementation*

1. How have you received information about Precision Health/your role in Precision Health? What additional information would you have liked? What is the best way for this information to be shared with you?
2. What would Precision Health need to include to scale to a whole pilot clinic? What about all SHC primary care?

*Patient Needs and Experiences*

1. How did you decide which patients to invite to Precision Health?
2. Which types of patients do you think Precision Health helped? Why? Are there patients you wouldn’t recommend it for?
3. Do you have any stories of patients who had good/bad/unexpected outcomes as a result of Precision Health?
4. For patients who are not comfortable with technology, can Precision Health help them to the same extent it might help technologically savvy people?
5. How did you manage patient concerns about Precision Health?
6. How did Precision Health impact your communication with patients?

*Diversity in Precision Health*

1. We are interested in how precision health may impact patients from low-income or racial/ethnic minority communities. How do you think Precision Health may need to be adapted or tailored to meet the needs of racial/ethnic minority or low-income patients?
2. We are also interested in how Precision Health has been received by diverse races/ethnicities. What is your perspective on how non-white patients have perceived Precision Health? How have you changed your practice to address non-white/European descent patient concerns or needs?

*The Future of Precision Health*

1. What are your thoughts about the sustainability of this approach to Precision Health? What would make Precision Health more sustainable?
2. What is your opinion about the future adoption of Precision Health by healthcare systems?
3. Are there other components of precision health that you would want to see added?

*Closing*

1. What suggestions do you have to improve Precision Health?
2. Is there something else about Precision Health and your experience that you would like to add? Something else we should have asked or that we should ask other providers?
