# Supplementary material for: Implementation outcomes of Humanwide: integrated precision health in team-based family practice primary care
Source: BMC Fam Pract. 2021 Feb 2;22:28. doi: 10.1186/s12875-021-01373-4 (PMC7856755; doi:10.1186/s12875-021-01373-4)
Supplement: Supplementary file 2 — Additional file 2. Precision Health Patient Interview Protocol. [file 12875_2021_1373_MOESM2_ESM.docx]

**Precision Health Patient Interview Protocol**

September 2018

| **Key Components**  Implementation  Pharmacogenetics  Genetic testing  Digital health/Health coaching | **Interview Goals**   - Patient experience - Patient involvement barriers and facilitators - Understand potential for implementation of Precision Health among racial/ethnic minorities |
| --- | --- |

**Protocol**

*Introduction/General*

1. What were your primary reasons for participating in Precision Health? How did they change over time?(3)
2. Would you recommend Precision Health to family members or friends? Why or why not? What would you say about your experience?
3. Have you finished your Precision Health experience? How did you know you had wrapped up? Did anyone tell you?
4. How do you think Precision Health will continue to impact your healthcare? Are there parts of Precision Health you will continue to do after completing the program?

*Knowledge/Beliefs/Attitudes*

1. Do you feel Precision Health was a good fit for you? What could have made your experience better?
2. How was your experience in each part of the Precision Health pilot different from what you expected when you first signed up? How was it the same as what you expected?
3. Which parts of Precision Health were most interesting or beneficial? Which parts were least interesting or beneficial? (As a reminder the components were: Health coaching, genetics testing, pharmacogenomics, and digital health).
4. How are the services and care provided through Precision Health different from primary care services you’ve experienced in the past?
5. How is the Precision Health team different from primary care teams you have interacted with in the past?
6. How has Precision Health impacted your health? To what extent do you think Precision Health has helped you meet your current health goals? What about future health goals?
7. How has participating in Precision Health impacted your day-to-day life?
8. Which Precision Health results were most valuable to you? Why?

*Diversity and Precision Health*

1. Some participants in a focus group, not anybody involved in this study, have mentioned concerns about precision health. Did you have any concerns when you started?
2. I’m going to list out 5 concerns people mentioned in the focus groups. Let me know if any of these seemed relevant to you:
   1. Privacy of your health information,
   2. The cost of genetic tests, and the cost of precision health overall,
   3. Different treatment from providers because of your race or ethnicity,
   4. Lack of trust in the healthcare system,
   5. Language barriers, and
   6. Physician knowledge of precision health.

What you would say to someone with these concerns?

*Closing*

1. What was the biggest surprise in participating in Precision Health? What was your biggest disappointment?
2. What suggestions do you have to improve Precision Health?
3. Is there anything else regarding Precision Health and your experience that you would like to add?

Thank you so much for your time!

If time: One more thing I wanted to follow-up on: Were there any parts you didn’t do? Why? (As a reminder the components were: Health coaching, genetics testing, pharmacogenomics, and digital health).
